# Can AI Bridge or Widen Maternal Health Inequities?

**DOI:** 10.1002/puh2.70119

**Published:** 2025-09-18

**Authors:** Reuben Victor M. Laguitan, Gilbert D. Bernardino

**Affiliations:** ^1^ Department of Medical Laboratory Science University of the Cordilleras Baguio Philippines; ^2^ College of Nursing University of the Cordilleras Baguio Philippines

**Keywords:** algorithmic bias, artificial intelligence (AI) in healthcare, health equity, low‐ and middle‐income countries (LMICs), maternal health

## Abstract

Artificial intelligence (AI) is rapidly transforming maternal healthcare through tools like risk prediction algorithms, telemedicine platforms, and postpartum support chatbots. Although these innovations offer promise, particularly in low‐ and middle‐income countries (LMICs), their impact on health equity remains contested. This commentary explores how AI can either bridge or widen maternal health inequities, depending on how it is designed, governed, and implemented. We introduce a conceptual framework comprising four interdependent domains that shape equity outcomes in maternal health: inclusive data practices, equitable governance, participatory design, and local capacity‐building. Drawing from interdisciplinary literature, we situate AI within broader health and social systems and argue for equity‐oriented approaches that foreground representation, accountability, and community engagement. By examining both opportunities and risks, this commentary offers practical, context‐sensitive recommendations for LMICs to ensure AI serves as a tool for justice in maternal healthcare.

## 1 Introduction

In 2020, approximately 287,000 women died from preventable pregnancy and childbirth‐related causes, which equates to nearly 800 deaths every day, with one maternal death occurring every 2 min [1, 2, 3]. Despite a 34% reduction in global maternal mortality between 2000 and 2020, progress has been uneven. The vast majority of maternal deaths continue to occur in low‐ and middle‐income countries (LMICs), where access to timely, high‐quality sexual, reproductive, and maternal health (SRMH) services remains limited [3]. Addressing these disparities is critical to achieving universal health coverage (UHC) and the Sustainable Development Goals (SDGs) [2].

As health systems explore digital transformation, artificial intelligence (AI) is emerging as a powerful tool in diagnostics, monitoring, and personalized care. In this commentary, AI refers to a range of technologies, including machine learning algorithms, clinical decision support systems, predictive analytics, large language models (LLMs), and chatbots, each of which plays a growing role in maternal healthcare.

Although early research on AI in digital health has often prioritized technical performance metrics such as diagnostic accuracy and model efficiency, there remains limited focus on the structural inequities and sociopolitical dynamics that shape real‐world outcomes [4].

Existing literature tends to overlook how these technologies interact with context‐specific challenges in maternal health, particularly in LMICs, where infrastructure, representation, and governance systems remain highly variable. Few studies adopt an explicitly equity‐centered lens that examines how AI may reproduce or reshape historical health disparities in these settings. This commentary addresses this gap by analyzing the structural, ethical, and sociotechnical factors that influence AI's impact on maternal health outcomes.

The term “data colonialism” also emerges in critical discussions, referring to the extraction and use of data from LMIC populations by external actors, often without reciprocal benefit, consent, or local control [5].

To address this analytical gap, this commentary employs a narrative synthesis of peer‐reviewed literature, WHO technical documentation, and global health policy analyses. Rather than presenting empirical findings, we integrate insights across disciplines to assess how structural, technological, and ethical conditions influence the role of AI in maternal healthcare. This analysis is guided by a conceptual framework that situates AI within a broader sociotechnical system, identifying four interdependent domains that influence whether AI applications contribute to equity or exacerbate existing disparities, particularly in LMICs.

## 2 Inclusive AI for Maternal Health: A Framework for Equity

**FIGURE 1:**
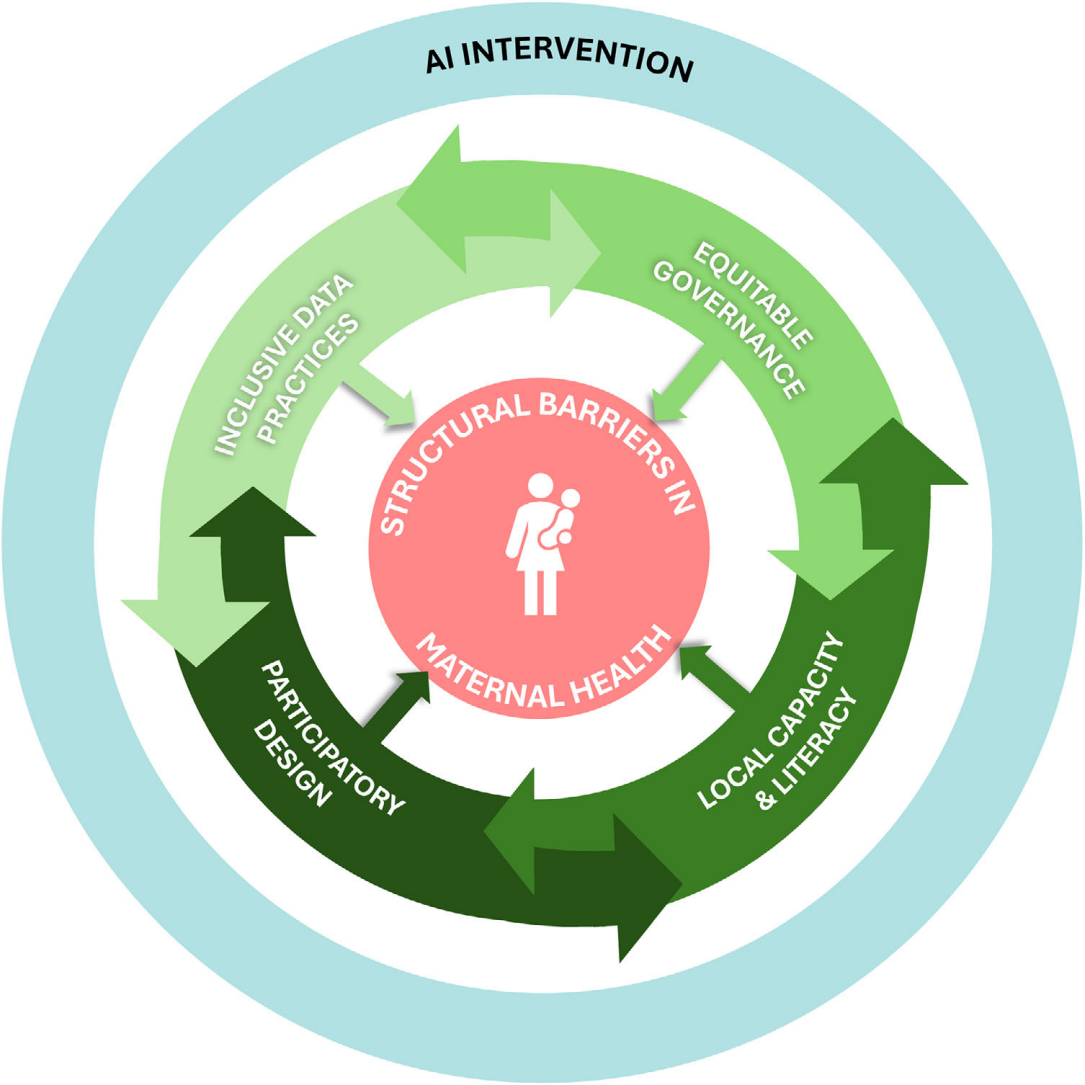
Conceptual framework for equitable AI in maternal health. AI, artificial intelligence.

The proposed framework comprises four interdependent domains that shape AI's equity outcomes in maternal healthcare: inclusive data practices, equitable governance structures, participatory design processes, and local capacity and digital literacy (please see Figure 1).

Inclusive data practices involve the systematic integration of diverse, representative datasets to reduce algorithmic bias and enhance clinical relevance across varied populations. Such practices are essential for minimizing racial, gender‐based, and regional biases that may otherwise compromise the reliability and safety of AI systems, especially in underrepresented populations [6, 7].

Equitable governance structures pertain to transparent and accountable mechanisms for overseeing data use, algorithmic decision‐making, and privacy protections, ensuring that power dynamics do not marginalize already underserved communities. These governance systems are particularly critical in LMIC settings where regulatory frameworks may be weak or unevenly enforced. Without robust oversight, AI technologies risk reinforcing existing hierarchies and structural inequities in health access and outcomes [8, 9].

Participatory design processes engage affected populations, especially women from historically disadvantaged groups, in the co‐creation and iterative evaluation of AI systems, thereby improving contextual fit and usability. This approach moves beyond user testing by embedding stakeholders throughout the design lifecycle, ensuring that tools reflect community values, preferences, and lived experiences [10, 11]. Participatory frameworks have been especially emphasized in LMIC contexts, where local norms, gender dynamics, and access constraints must be factored into the technology design process [12].

Finally, local capacity and digital literacy encompass the infrastructural, institutional, and educational foundations necessary for communities to actively engage with AI technologies, rather than remain passive recipients of externally imposed tools. In LMIC settings, this includes strengthening digital infrastructure, building institutional capacity to maintain AI systems, and training healthcare workers to responsibly interpret and interact with AI tools. Recent scoping reviews emphasize that without these foundational capacities, AI systems risk becoming underutilized, mistrusted, or misaligned with local needs, and that successful AI integration depends not only on technology availability but on long‐term investments in human capital, digital fluency, and local ownership of AI‐enabled health systems [13, 14].

These domains do not function in isolation; they operate as both mediators and predictors of whether AI will serve as a tool for equity or reinforce structural barriers. If these domains are deliberately strengthened, AI technologies can help bridge long‐standing service gaps, improve the continuity of maternal care, and reduce preventable maternal deaths. If ignored, however, they may lead to biased outputs, unregulated deployment, and deepening forms of digital exclusion.

This conceptual framework forms the analytical backbone of the commentary. It will be used throughout the analysis to evaluate how emerging AI interventions interact with health systems, governance, and community engagement. The dynamic relationship among these domains is illustrated in Figure 1.

To contextualize this framework, it is necessary to examine the deep‐rooted maternal health inequities that persist globally, especially in LMICs where these technologies are increasingly being introduced.

## 3 The Persistent Challenge: Maternal Health Inequities

Despite notable progress over the past two decades, maternal health outcomes remain profoundly unequal between high‐income countries (HICs) and LMICs. In 2023, approximately 260,000 women died from preventable causes related to pregnancy and childbirth, which equates to one maternal death every 2 min. Alarmingly, 92% of these deaths occurred in low‐ and lower‐middle‐income countries, with sub‐Saharan Africa alone accounting for nearly 70% of all maternal deaths [3].

These inequities are rooted in persistent structural barriers such as inadequate access to skilled birth attendants, emergency obstetric care, and essential health technologies. In many LMICs (especially in rural and conflict‐affected areas), health facilities are understaffed and under‐resourced, lacking electricity, clean water, transportation, and trained personnel [15]. Additionally, out‐of‐pocket healthcare expenses remain a major obstacle, discouraging timely care‐seeking behavior among pregnant women in resource‐constrained environments.

UHC aims to ensure that all individuals receive quality healthcare without financial hardship. However, SRMH services remain among the most under‐resourced aspects of primary healthcare systems in LMICs. Many women are still unable to access essential services (e.g., antenatal checkups, safe childbirth assistance, contraception, and postpartum care), undermining both maternal health outcomes and the broader goal of achieving SDG 3.1 [2, 16].

In some regions, additional social and legal factors further intensify these inequities. In Latin America and the Caribbean, unintended pregnancies and unsafe abortions remain major contributors to maternal morbidity and mortality. Adolescents and women from marginalized communities are especially at risk due to restrictive abortion laws, limited access to contraception, and stigma around reproductive health services [15].

These structural barriers are compounded when maternal health risks are shaped by overlapping disadvantages such as age, ethnicity, socioeconomic status, and geographic location [17]. For instance, adolescent mothers from indigenous or marginalized communities may face a combination of stigma, limited access to contraception, inadequate transportation, and language barriers. Similarly, older women from underserved ethnic groups may encounter delayed care, mistrust from providers, and implicit bias in clinical settings. These intersecting factors deepen vulnerability and restrict access to timely and respectful care. If AI systems are developed without accounting for such layered inequities, they risk reproducing the very disparities they aim to address. This highlights the critical need for inclusive data practices and participatory design, which are central to our conceptual model for advancing equity in maternal health.

Addressing these systemic barriers is essential to closing the maternal health gap and ensuring that emerging technologies, such as AI, do not replicate but rather help dismantle the structural inequities that have long shaped maternal health outcomes.

Recognizing these challenges, the following section explores how AI, when thoughtfully designed and implemented, can serve as a powerful tool to address the gaps in maternal healthcare.

## 4 Opportunities: How AI Can Potentially Bridge Gaps in Maternal Healthcare

AI is revolutionizing maternal healthcare by addressing critical gaps in access, quality, and continuity of care, especially in low‐resource settings. Rather than functioning as isolated tools, AI applications work synergistically across multiple layers of maternal health delivery, offering integrated support for both patients and providers.

At the point of care, AI enhances early risk detection and personalized health management. Machine learning algorithms analyze patient history, vital signs, and biometric data from wearable technologies to identify complications, such as preeclampsia or gestational diabetes, before they escalate, enabling timely interventions in contexts where diagnostic tools are scarce [18]. These predictive capabilities are further bolstered by AI‐enabled screening models that identify risk profiles for outcomes like stillbirth, offering a proactive approach to obstetric risk management [19].

Emerging evidence from LMICs illustrates how AI‐powered and digital tools can improve maternal health outcomes when adapted to local needs and health system capacities. In Ghana, a cross‐sectional study found that mobile health (mHealth) technologies were widely used by healthcare professionals to support diagnosis, treatment, and communication, contributing to improved service delivery in maternal health settings [20]. This uptake reflects how localized technological capacity and digital fluency among providers can shape the effective deployment of AI, especially in settings where human infrastructure often determines a tool's success more than the algorithm itself.

In India, the REAN Foundation developed an AI‐powered chatbot that enables non‐invasive screening for anemia, a major risk factor in pregnancy. This tool allows for earlier identification and intervention among women in resource‐constrained communities [21]. By addressing a pregnancy‐specific condition through regionally adapted tools, this initiative reflects the value of contextually relevant data and design, underscoring how tailored inputs and user‐centered development can enhance equity in AI outcomes.

Meanwhile, in Uganda, a Gates Foundation‐supported initiative deployed AI‐assisted portable ultrasound devices in rural health centers, empowering midwives to detect obstetric complications and make timely referrals without requiring specialists on site [22]. Equipping midwives to interpret and act on AI outputs without relying on external specialists illustrates the importance of strengthening human capacity at the community level, especially in settings where traditional healthcare infrastructure is limited. These examples show that AI, when integrated into supportive systems and governed ethically, can effectively address persistent barriers in maternal healthcare, including limited access, delayed risk detection, and inadequate diagnostic support in underserved areas.

The integration of AI into remote and telehealth services redefines care delivery across distances. AI‐supported telemedicine platforms enable personalized consultations and continuous monitoring, making specialized care accessible to women in geographically isolated or underserved communities [23]. These applications, when co‐developed with grassroots stakeholders, are more likely to respect local care norms and accessibility needs. This underscores the often overlooked yet essential role of inclusive design in fostering trust and usability across diverse populations.

Beyond clinical care, AI‐powered chatbots and mobile‐based interventions have emerged as valuable channels for health education, mental health support, and postpartum guidance. When thoughtfully designed, such tools can reflect the needs and experiences of their users, particularly by providing nonjudgmental and anonymous support to women experiencing mood and anxiety disorders. This, in turn, encourages early help‐seeking behavior [24]. Their effectiveness, however, is contingent upon participatory design processes and the local capacity to access and engage with digital platforms.

AI‐enhanced systems deliver real‐time diagnostic support and treatment guidance, enabling frontline health workers to navigate complex clinical scenarios with greater confidence and precision, especially in settings where specialist availability is limited [25]. However, their reliability depends heavily on the quality of the datasets that underpin them. This underscores the importance of inclusive and representative data collection as a foundation for clinical accuracy and equity. For example, telemedicine and digital health interventions have been deployed to expand access to maternal care in Africa, illustrating the potential of these technologies to overcome geographical and economic barriers in low‐resource environments [26].

Beyond individual patient care, AI optimizes the functioning of maternal health systems. From managing supply chains for essential medicines to analyzing population‐level data for epidemiological surveillance, AI enables more efficient resource allocation and early detection of public health threats [23]. In‐home care models are also evolving, with AI coordinating remote postpartum support and care planning to ensure continuity beyond the health facility [27].

Collectively, these advancements demonstrate AI's vast potential to bridge maternal health inequities if deployed ethically and inclusively. When aligned with local needs and supported by adequate infrastructure, AI can serve as a powerful equalizer in the global pursuit of safer pregnancies and healthier mothers. Although the applications above demonstrate AI's significant potential to address maternal health gaps, their effectiveness is not guaranteed. The outcomes of AI deployment are shaped by systemic factors, including governance, representation, infrastructure, and ethical oversight, all of which influence whether these tools are accessible, equitable, and sustainable. Without deliberate attention to these broader conditions, well‐intentioned AI interventions may fail to reach those most in need or, worse, reinforce the very disparities they aim to resolve.

These examples make it clear that technological innovation, although important, is not sufficient on its own. The success of AI tools in maternal healthcare depends on their alignment with local health system realities, cultural norms, and community capacities. Engagement with frontline workers, attention to social context, and adaptation to digital literacy levels are all critical to ensuring meaningful and lasting impact. When these enabling factors are absent, even the most promising technologies may fail to produce equitable outcomes. The following section critically examines these underlying structural and ethical dimensions.

## 5 Strategies for Responsible, Equitable, and Inclusive Implementation

For AI to serve as a bridge rather than a barrier in maternal healthcare, its development and deployment must be rooted in equity, ethical governance, and local empowerment. Achieving this requires deliberate, systemic strategies that move beyond technological optimism and confront the structural realities shaping healthcare access and outcomes, especially in LMICs.

At the core of responsible AI lies the principle of inclusive and representative data. This issue illustrates how algorithmic performance is inextricably tied to equity: When inclusive data practices are not prioritized, AI systems risk reproducing harm rather than mitigating it. Without proactive efforts to diversify data by race, gender, geography, and socioeconomic status, such systems risk reinforcing the very inequities they purport to solve [28, 29]. More than a technical flaw, this exclusion is an ethical failure, one that perpetuates structural bias under the guise of neutrality.

A genuinely equitable AI approach demands the active participation of the communities it intends to serve. Embedding end‐users into the design process helps ensure that tools are relevant, trustworthy, and responsive, an approach essential to reducing maternal health disparities. Women, especially from historically marginalized groups, must be involved not just as subjects of research but as co‐designers and evaluators of AI tools. Participatory design methodologies, which engage users in shaping technologies, have been shown to increase the relevance, usability, and acceptance of digital health interventions [10]. In maternal health, such engagement is vital to ensure that AI reflects the lived experiences of those navigating pregnancy, birth, and postpartum care under diverse and often precarious conditions.

Ethical deployment also hinges on the establishment of clear governance frameworks. In LMIC settings, where regulatory systems may be weak or evolving, these structures must not only ensure safety but also actively redistribute decision‐making power, ensuring equity is embedded at the policy level. These must include enforceable standards for data privacy, consent, accountability, and transparency. Regulatory bodies should define who is responsible when AI outputs lead to harm and ensure that algorithms remain auditable and explainable. Moreover, these frameworks must evolve with technology, enabling real‐time oversight rather than relying solely on retrospective reviews [8]. The equity of AI systems depends on the strength of the domains outlined in our conceptual model: data practices, governance, design, and local capacity, each of which requires deliberate ethical and political attention.

Ethical challenges in AI go beyond abstract principles and manifest in real clinical harm when equity is neglected. For example, several maternal health prediction models, which were trained predominantly on datasets from white, Western populations, have demonstrated reduced accuracy in detecting preeclampsia and other hypertensive disorders among African and Afro‐descendant women [23]. This disparity is not simply a data gap but an ethical failure rooted in the exclusion of diverse populations from the design and validation process. Responsible AI demands more than algorithmic efficiency; it requires informed consent processes that clearly communicate how data will be used, data sovereignty to ensure that LMIC communities maintain control over their information, and algorithmic accountability so that errors can be traced, audited, and corrected. Without these safeguards, AI may inadvertently deepen systemic injustices, reinforcing existing disparities through mechanisms that appear neutral or innovative on the surface.

Another central consideration is the ongoing evaluation and auditing of AI systems. Too often, tools are deployed without sufficient testing in real‐world settings, especially in LMICs. Continuous monitoring for bias, inequity, and performance disparities is essential, not only to ensure safety but also to maintain public trust. Feedback loops involving both healthcare providers and patients are critical to refining these tools in practice, rather than relying exclusively on theoretical design principles [30].

Equity also depends on educational investment. When users understand how AI systems work, they are more empowered to question, adapt, or resist harmful outputs, making literacy an essential condition for local autonomy and ethical innovation. Healthcare professionals must be equipped with training to interpret and use AI responsibly, and communities must be informed of their rights regarding data use, consent, and health decision‐making. AI literacy tailored to diverse linguistic, cultural, and digital access levels can empower users to engage critically with these technologies, rather than be passively shaped by them [31].

Crucially, the capacity to design, manage, and maintain AI tools must be cultivated locally. Investing in AI education and infrastructure in LMICs not only supports sustainability but also ensures that technologies are not only sustainable but also accountable to the communities they intend to serve. Local ownership helps tailor tools to specific health systems and sociocultural realities and counteracts the asymmetries of “data colonialism,” in which external actors extract data and value without reciprocal benefit to the communities being mined [5].

Despite growing interest in AI for health, many LMICs face persistent challenges in building and sustaining local capacity. One major barrier is brain drain, as skilled professionals often seek opportunities abroad due to limited career prospects, infrastructure, or compensation at home [32]. Additionally, funding for local AI development and education is frequently fragmented or reliant on short‐term grants, raising concerns about long‐term sustainability [33]. Addressing these challenges requires not only investment in training and infrastructure but also sustained commitments from governments, academic institutions, and international partners to foster environments where skilled professionals can remain, thrive, and lead innovation from within their communities. Achieving true equity means building from within, not imposing solutions from outside.

Lastly, achieving responsible AI in maternal health will require cross‐sector collaboration and new models of funding. Governments, NGOs, academic institutions, and private industry must co‐develop funding mechanisms and implementation roadmaps that prioritize inclusivity. Global standards‐setting bodies must also open seats at the table for LMIC voices, not merely as participants, but as agenda‐setters. Only through inclusive governance can AI evolve into a truly global health technology.

**FIGURE 2:**
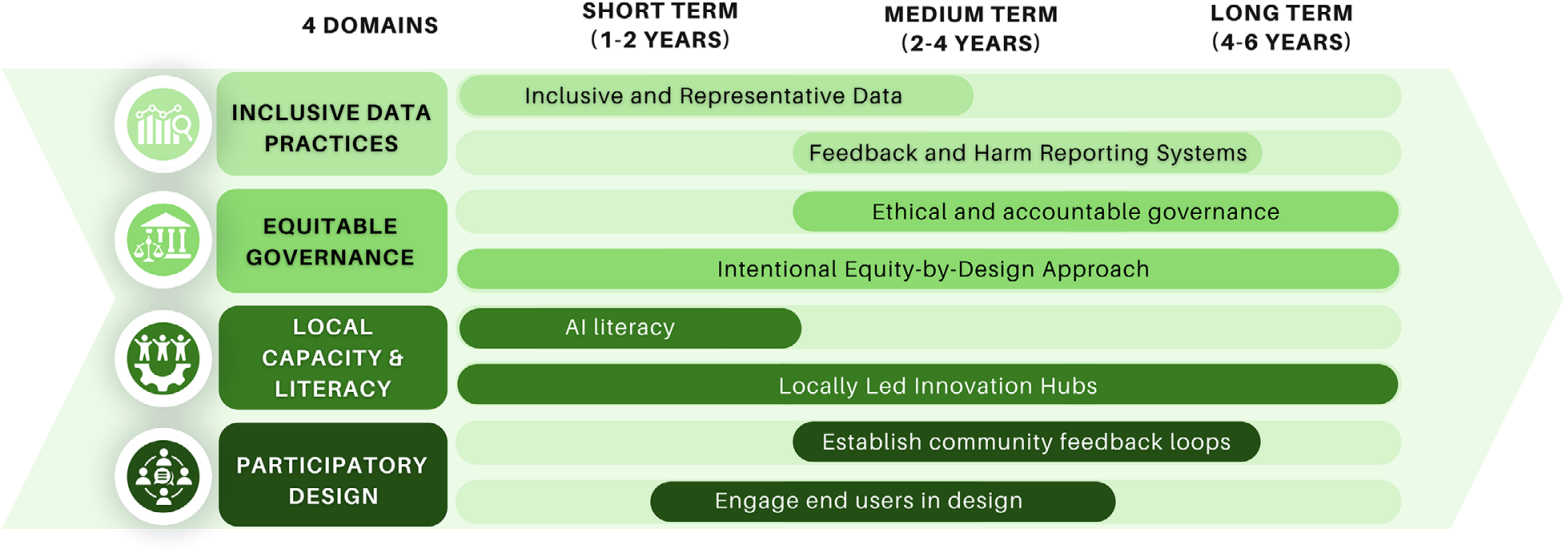
Roadmap for Equitable AI Implementation in Maternal Health. AI, artificial intelligence. *Source:* Timeline categories are adapted from WHO's Global Strategy on Digital Health 2020–2025 [34].

To operationalize the ethical and equity‐focused vision of AI in maternal healthcare, a practical roadmap is needed, one that translates principles into concrete actions. The Roadmap for Equitable AI Implementation in Maternal Health outlines a time‐bound, phased strategy comprising short‐term (1–2 years), medium‐term (2–4 years), and long‐term (4–6+ years) priorities (Figure 2). These align with global health equity goals and the lived realities of women and communities in LMICs.

In the short term (1–2 years), there must be sustained investment in locally led AI research centers in LMICs. However, before establishing such hubs, the initial phase must focus on laying a strong foundation of understanding and representation.

Central to this short‐term phase is the promotion of AI literacy and public education. Educational efforts must target both healthcare providers and patients to demystify AI tools and promote informed engagement. Culturally appropriate campaigns and user‐friendly toolkits can foster trust, reduce fear, and empower communities to interact meaningfully with AI in maternal health.

Global standards should mandate the inclusion of diverse and representative datasets, ensuring that AI systems are trained on data reflective of racial, geographic, and socioeconomic variation. Mandating inclusive datasets and conducting early data audits to detect and correct bias are short‐term priorities. Without inclusive data, AI risks becoming a partial lens that misrepresents the populations it intends to serve.

In the medium term (2–4 years), true equity also requires participatory design processes that meaningfully engage women, frontline health workers, and local stakeholders in co‐creating the tools intended to serve them. AI tools should be developed in close collaboration with these groups through co‐design and iterative user testing in real‐world maternal care settings. These design efforts must be supported by ongoing data updates and audits to ensure continued representativeness over time.

Ethical implementation further depends on enforceable governance standards, including requirements for informed consent, algorithmic transparency, explainability, and data sovereignty. During the medium‐term phase, national ethical review bodies should be strengthened to provide oversight, ensuring the safe and fair application of AI across diverse maternal health contexts.

To support meaningful engagement, AI literacy campaigns must be launched to equip users with the capacity to question and shape AI systems.

Also in the medium‐term phase, community‐based feedback and harm reporting systems should be introduced. These mechanisms allow for ongoing monitoring of AI's equity and safety impacts, enabling timely redress in cases where harm occurs. These systems act like a tuning mechanism to ensure AI tools remain responsive and just.

In the long term (4–6+ years), the roadmap prioritizes the establishment and funding of locally led innovation hubs in LMICs. These hubs will serve as regional centers for AI research and development rooted in local maternal health priorities, enabling adaptation, validation, and continuous evaluation of maternal health AI tools. This supports sustainability, cultural relevance, and community ownership of AI innovations.

Throughout all phases, the roadmap emphasizes global equity and solidarity. It calls for fostering international collaborations in AI research, governance, and funding. These partnerships aim to ensure that LMIC‐led initiatives are integrated into global innovation ecosystems, promoting a fairer distribution of technological benefits.

Taken together, these phased, interlinked actions form a structured and accountable pathway toward AI advancement that is both technologically robust and socially just. Each step maps onto one or more domains of our conceptual framework, providing an actionable translation of its principles. Embedded across every stage of the roadmap is a commitment to equity‐by‐design, a guiding principle ensuring that equity is systematically embedded, assessed, and upheld throughout the development, deployment, and evaluation of AI systems.

Ensuring that AI serves to narrow, rather than widen, the persistent inequities in maternal health demands more than innovation; it demands intention. This means embedding equity at every stage of AI development and deployment. Intentional design must prioritize the needs of marginalized communities, avoiding algorithmic biases that could exacerbate disparities. Community inclusion is vital, not as an afterthought, but as a foundational principle that will ensure that the voices of women, especially those from underserved populations, shape the technologies intended to support their care. Furthermore, ethical regulation must go beyond technical compliance to actively protect human rights, privacy, and the inherent dignity of individuals. Lastly, global solidarity is required to share resources, knowledge, and advocacy across borders, recognizing that maternal health inequities are not confined by geography but are part of a broader global justice issue.

This is not merely a technical challenge to be solved with better code or more data, but it is a moral imperative that compels us to ask: *Who is AI serving, and at whose expense?* To truly advance equity in maternal health, we must treat the integration of AI not as a neutral process but as one that reflects and reinforces societal values. Only by confronting these ethical dimensions head‐on can AI become a tool for justice rather than a mechanism of further exclusion.

## 6 Conclusion

AI holds transformative potential in maternal and reproductive health only when designed and deployed with equity at its core. Its power to predict, personalize, and extend care is significant, especially in low‐resource settings, yet these benefits are not automatic. The trajectory of AI's impact depends entirely on how its systems are governed, who participates in their design, and whether local realities shape their development from the ground up.

To ensure AI contributes meaningfully to maternal health equity, it must be integrated into a broader global health justice agenda aligned with the principles of UHC and the SDGs. This calls for coordinated investment in inclusive data governance, participatory research, and capacity‐building at the local level. Continuous evaluation, algorithmic accountability, and the involvement of multidisciplinary stakeholders, including women, frontline health workers, and LMIC policy leaders are essential to ensuring both technical soundness and ethical legitimacy.

AI alone will not transform maternal health. It is the values embedded within its design, governance, and deployment that will ultimately determine its true impact. Whether it empowers or excludes depends on whose voices shape its development, whose data informs its logic, and whether historically marginalized communities are positioned as co‐creators rather than passive recipients.

As a conceptual commentary, this article does not present new empirical data but instead synthesizes existing literature to propose a framework for equity‐oriented AI in maternal health. Future research should explore the implementation of this framework through case studies, participatory pilot programs, and policy evaluations in diverse LMIC contexts. Empirical work is also needed to assess the impact of inclusive data practices, community‐driven design processes, and local capacity‐building efforts on maternal health outcomes and AI system performance. Comparative studies across regions could further identify which equity strategies are most effective under varying resource and governance conditions.

The path forward demands not just innovation but a steadfast commitment to justice, inclusion, and systemic equity.

## Author Contributions

**Reuben Victor M. Laguitan**: conceptualization, methodology, writing – original draft, writing – review and editing, investigation, literature review, final approval. **Gilbert D. Bernardino**: literature review, writing – review and editing, supervision, final approval.

## Ethics Statement

The authors have nothing to report.

## Conflicts of Interest

The authors declare no conflicts of interest.

## Data Availability

No data were generated or analyzed for this commentary.
